# Enrichment of Hyaluronic Acid Binding Tumor Cells by Modulation of Selective Adhesion on Microgel Surfaces

**DOI:** 10.1002/marc.202500549

**Published:** 2025-09-24

**Authors:** Melanie Schmidt, Dilay Karayel, André Franken, Janita Müller, Laura Hartmann, Klaus Pantel, Tanja Fehm, Hans Neubauer, Stephan Schmidt

**Affiliations:** ^1^ Department of Obstetrics and Gynecology University Hospital and Medical Faculty of the Heinrich‐Heine University Duesseldorf Düsseldorf Germany; ^2^ Center for Integrated Oncology (CIO Aachen, Bonn, Cologne) Duesseldorf Germany; ^3^ Faculty of Mathematics and Natural Sciences Institute of Organic Chemistry and Macromolecular Chemistry Heinrich‐ Heine‐ University Düsseldorf Düsseldorf Germany; ^4^ Department for Tumor Biology University Medical Centre Hamburg‐Eppendorf Hamburg Germany; ^5^ Institute for Macromolecular Chemistry Faculty of Chemistry and Pharmacy Albert‐Ludwigs‐Universität Freiburg Freiburg Germany

**Keywords:** cell sheet, circulating tumor cells (CTC), hyaluronic acid (HA), LCST, liquid biopsy, lower critical solution temperature, MDA‐MB‐231 breast cancer cells, Poly(N‐isopropylacrylamide) (PNIPAM), smart polymers, thermo‐responsive material, volume phase transition temperature (VPTT)

## Abstract

Liquid biopsy‐based detection of cells with specific biomarker profiles is critical for cancer diagnostics and treatment. Here, we present a novel method for the selective enrichment of CD44‐expressing tumor cells from blood using thermoresponsive microgel surfaces functionalized with hyaluronic acid (HA). A key feature of our approach is the precise modulation of CD44–HA interactions through the microgels’ volume phase transition temperature (VPTT). Lowering the temperature from 37°C to 30°C induces swelling of the microgel layer, thereby diminishing adhesive interactions and promoting the detachment of weakly adhering white blood cells (WBCs), while strongly adherent tumor cells remain captured. Flow cytometry analysis studies further reveal that tumor cells with elevated CD44 expression exhibit persistent adhesion on HA‐functionalized surfaces. Given the straightforward fabrication process and the versatility for incorporating various biomarkers via chemical synthesis, this temperature‐responsive microgel platform holds promise for the efficient capture of circulating tumor cells (CTCs) present in the blood of cancer patients and other challenging diagnostic applications.

## Graphical abstract

We have developed a thermoresponsive microgel system for the selective enrichment of hyaluronic acid‐binding tumor cells. By modulating the adhesion on the microgel surface via temperature changes from 37°C to 30°C, we demonstrate a novel proof‐of‐principle for separating these tumor cell from white blood cells, which has potential applications in diagnostics and therapeutics.

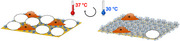

## 1 Introduction

Responsive surfaces have been extensively explored in the past decades for applications in cell culture, tissue engineering, and regenerative medicine [1, 2, 3]. In particular, thermoresponsive polymers such as poly(N‐isopropylacrylamide) (PNIPAM) have been widely used to create switchable surfaces that enable cell adhesion and detachment in a cell‐friendly manner [4, 5, 6]. The pioneering work by Okano and others demonstrated that cell sheets could be harvested from PNIPAM‐coated surfaces simply by reducing the temperature, making such systems highly attractive for biomedical applications [7]. More recently, microgel‐based coatings have emerged as a versatile alternative to thin grafted polymer films, offering advantages such as simple fabrication, increased mechanical robustness, and tunable properties via chemical functionalization [8, 9, 10].

While responsive coatings have primarily been utilized for cell cultivation, they also hold significant potential for diagnostic applications. For instance, thermoresponsive polymer materials can reversibly present or conceal specifically interacting motifs, thereby modulating their adhesion strength to certain cell types. This has been demonstrated, for example, with carbohydrate‐functionalized PNIPAM‐based thermoresponsive polymers and microgels [11, 12, 13, 14, 15]. These systems exploit the fact that PNIPAM chains undergo a reversible phase transition at their critical temperature of approximately 32°C, switching between a collapsed globule and an extended coil conformation. In the collapsed state, the specifically interacting motifs are typically presented at a higher density on a more rigid surface, with reduced steric hindrance compared to the swollen state. As a result, for hydrophilic carbohydrate motifs an affinity enhancement for their cell target interactions is observed.

Therefore, the selective capture and controlled release of specific cell types is possible via temperature stimulus, which could enable improved liquid biopsy techniques, facilitate the isolation of CTCs, and aid in fundamental studies of cell adhesion and metastasis. The isolation and detection (CTCs) is important for early cancer diagnosis, and treatment monitoring. However, the rarity of CTCs amidst a vast background of hematologic cells poses significant challenges for their effective capture. Previous approaches for CTC enrichment often target epithelial cell surface proteins like EpCAM (epithelial cell adhesion molecule) [16, 17] as well as various additional cues and methods [18, 19, 20, 21, 22]. However, such methods may miss CTCs that have undergone epithelial‐to‐mesenchymal transition (EMT), leading to reduced EpCAM expression. To overcome these limitations strategies focusing on the depletion of WBCs to enrich the CTC population have gained attention. There are methods employing magnetic beads functionalized with anti‐CD45 antibodies, which effectively deplete WBCs from blood samples while preserving CTCs [23], or combine magnetic bead‐like chain structures within a microfluidic device [24, 25]. Filtration‐based methods also offer CTC isolation by exploiting size and deformability differences between CTCs and WBCs [26]. Additionally, hybrid approaches combining filtration with CD45‐ and avidin‐based immunodepletion achieved tumor cell capture with reduced WBC co‐capture [27]. Despite these advancements, challenges persist in balancing CTC recovery with WBC depletion.

One widely studied biomarker for tumor cell targeting is CD44, a major receptor for hyaluronic acid (HA) that is overexpressed in many cancer types, including breast cancer [28, 29, 30]. As a result, HA‐functionalized surfaces have been used to selectively capture CD44‐positive cells, allowing for the enrichment of tumor cells from complex biological samples [31, 32, 33]. However, despite the promise of HA‐based capture systems, challenges remain in achieving high specificity. A significant fraction of non‐tumor cells adheres to capture systems, as CD44 expression is not exclusive to tumor cells and is also present on various normal cell types, including WBCs [34].

**FIGURE 1:**
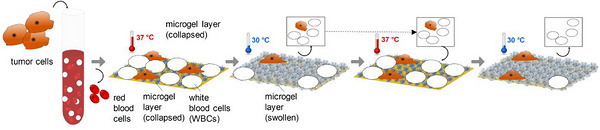
Temperature‐controlled enrichment of CD44‐expressing tumor cells using HA‐functionalized microgel surfaces. Tumor cells are spiked into whole blood and after erythrocyte lysis applied onto a hyaluronic acid (HA)‐decorated, thermoresponsive microgel surface at 37°C, where both tumor cells and white blood cells (WBCs) initially adhere. Upon cooling to 30°C, the microgel swells, leading to a decreased HA ligand density and selective detachment of weakly adhering WBCs, while strongly adherent CD44‐positive tumor cells remain attached. This reversible switching process is supposed to enable repeated (relative) enrichment and release for cell capture and analysis.

Therefore, a system is required that amplifies the binding of cells with strong receptor overexpression while minimizing the retention of weakly adherent cells. To address this issue, we introduce a dynamic cell sorting approach based on cyclic switching of adhesive interactions. This approach exploits thermoresponsive PNIPAM microgels functionalized with HA, where repeated cycles of cell adhesion and release progressively enrich the population of strongly adhering cells. We hypothesize that subjecting cells to multiple binding–release cycles will preferentially detach weakly adherent cells, thereby increasing the ratio of bound cancer cells to background WBCs. In this study, we investigate the efficiency of this cyclic sorting mechanism by comparing single‐ and multi‐cycle adhesion‐release experiments (Figure 1). As a model, we use MDA‐MB‐231 cells, which belong to the triple‐negative breast cancer subtype, that are clinically challenging with currently only few effective capture strategies. In addition, we also test a patient‐derived CTC‐ITB‐01 cell line [35]. We evaluate cell adhesion selectivity, release efficiency, and the extent of tumor cell enrichment under different experimental conditions. Our findings demonstrate that repeated binding‐release cycles significantly improve the specificity of HA‐based capture systems, offering a promising strategy for the isolation of rare tumor cells from complex biological samples such as blood.

## 2 Results and Discussion

### 2.1 Microgel Synthesis and Surface Preparation

**SCHEME 1:**
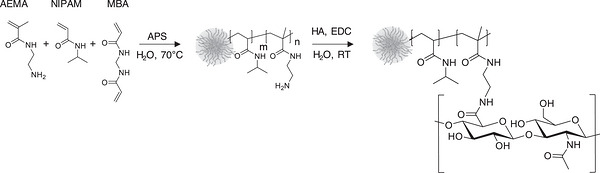
Synthetic steps toward the switchable AEMA‐HA microgels. Microgels were prepared via radical copolymerization of NIPAM, AEMA (cationic monomer), and MBA (crosslinker), followed by HA conjugation via EDC coupling. Heparin and cGM were conjugated to the AEMA1% microgels in the same way.

The microgels were synthesized via copolymerization using NIPAM as the primary monomer, with 1 mol% and 2 mol% AEMA (N‐(2 aminoethyl)methacrylamide hydrochloride) as an amine‐functionalized comonomer, and MBA (N,N methylenbisacrylamide) as a bifunctional crosslinker, initiated by ammonium peroxide by raising the temperature to 70°C. This single‐step radical copolymerization reaction results in the precipitation of precursor particles, which continue to grow into monodisperse microgels. The AEMA functionalized microgels decorated with just the cationic comonomer, AEMA1% and AEMA2%, are used as a negative control. Following purification through centrifugation and washing, the microgels were functionalized with HA via carbodiimide chemistry. Two different HA functionalized microgels were prepared AEMAl%‐HA AEMA2%‐HA (Scheme 1). In previous work, we identified that a HA functionalization degree of 50 µmol per gram microgel to be suitable for adhering MDA‐MB‐231 cells, whereas negative controls without HA functionalization lead to significantly reduced adhesion [36]. HA functionalization degrees of 22 and 76 µg mol^−1^ (Table 1) are consistent with previous work [14, 15, 36, 37]. As additional control samples, microgels functionalized with heparin and carboxylated galactomannan (cGM) were prepared (AEMA1%‐Heparin AEMA1%‐cGM). These controls were selected because heparin is another common glycan presented on the cell surface [38] and cGM presents galactose units that may also have interactions with tumor cells [39, 40].

**TABLE 1:** Microgel's hydrodynamic diameters, at 20°C, 30°C, and 40°C (*D_h_*_20_, *D_h_*_30_ *, D_h_*_40_) the microgel zeta potential (*ζ*), their degree of carbohydrate functionalization expressed as moles of hexoses per gram microgel.

Sample	*D_h20_* (nm)	*D_h30_* (nm)	*D_h40_* (nm)	*ζ_30_* (mV)	carbohydrate conc.^a^ (mmol g^−1^)
AEMA1%	369 ± 12	321 ± 5	172 ± 3	10.5 ± 0,5	n.a.
AEMA2%	310 ± 10	280 ± 7	163 ± 6	9.9 ± 1.5	n.a.
AEMAl%‐HA	518 ± 32	390 ± 10	241 ± 7	−5,1 ± 0,2	22 ± 11
AEMA2%‐HA	891 ± 25	720 ± 27	346 ± 3	−19.2 ± 2.3	76 ± 18
AEMA1%‐Heparin	915 ± 10	783 ± 12	470 ± 3	n.a.	102 ± 15
AEMA1%‐cGM	795 ± 40	474 ± 4	447 ± 17	−8.4 ± 0.8	48 ± 14

^a^ determined by the phenol‐sulfuric acid assay.

**FIGURE 2:**
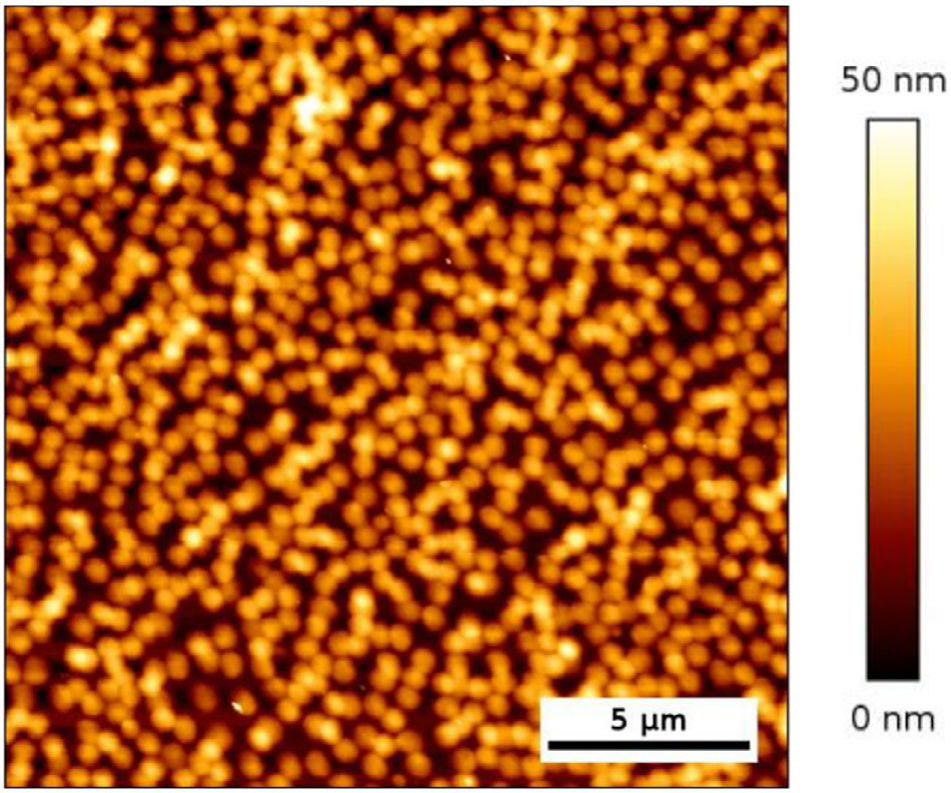
Atomic force microscopy (AFM) image of a dried monolayer of PNIPAM microgels deposited on a hydrophobized glass substrate. Note, that due to drying and lateral deswelling the packing of the film appears incomplete, whereas complete coverage is expected upon swelling in solution. The image confirms the formation of a uniform, close‐packed microgel layer indicating stable surface attachment. Scale bar: 5 µm.

Next, close‐packed monolayer microgel coatings were generated on hydrophobized glass surfaces to ensure sufficient adhesion between the microgels and the substrate in aqueous solution. Previous studies on PNIPAM microgel coatings in hydrophobic plastic dishes demonstrated that the binding remains stable even after mechanical agitation, rinsing, and similar treatments [12]. To create a monolayer of microgels, a straightforward drop‐casting technique was used, followed by drying and washing to eliminate excess microgels. When a surface is fully covered with a microgel dispersion at concentrations of 10 µg mL^−1^ or higher, densely packed monolayers are formed (Figure 2). After washing, the resulting microgel monolayers exhibited uniformity and remained stable throughout the cell assay, including washing steps and temperature changes. Consequently, this simple deposition method produces stable and well‐ordered microgel coatings.

### 2.2 Microgel Characterization

**FIGURE 3:**
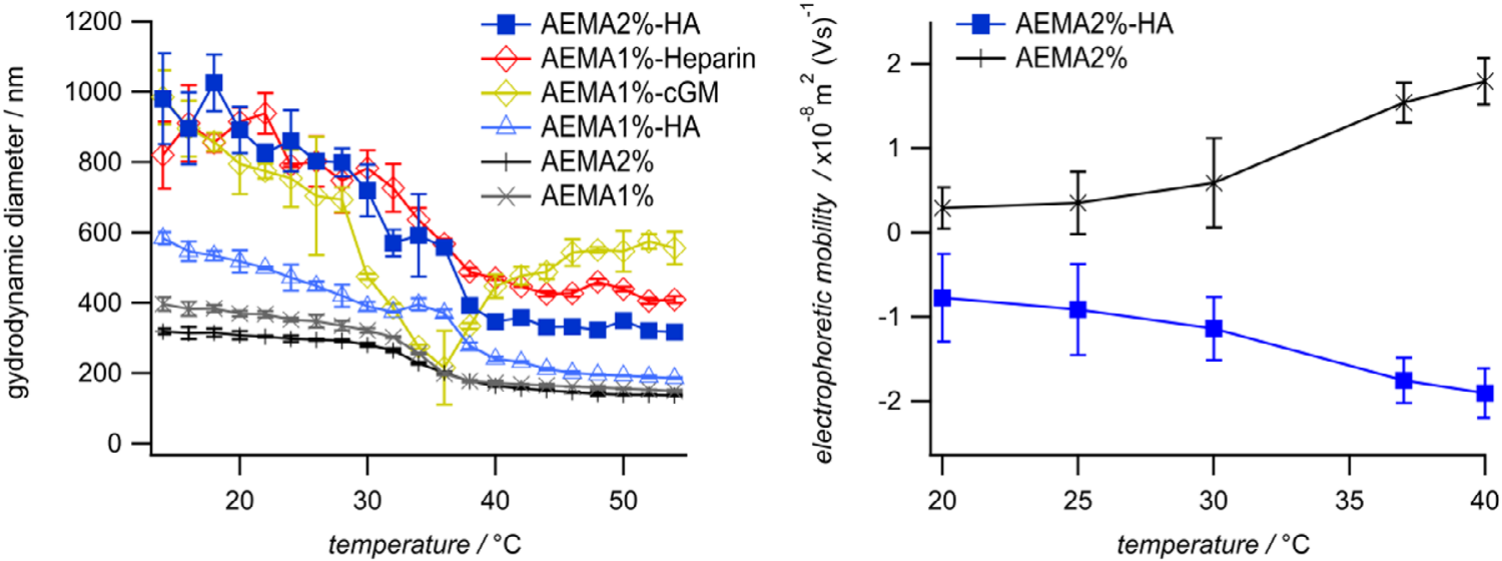
Thermoresponsive behavior and surface charge of AEMA‐based and glycan functionalized microgels. Left: Temperature‐dependent hydrodynamic diameters measured by dynamic light scattering (DLS) for microgels with different glycan modifications (HA, heparin, cGM) and AEMA contents. All microgels exhibit a size decrease around the VPPT. Right: Electrophoretic mobility of AEMA2% and AEMA2%‐HA microgels as a function of temperature.

To fine‐tune cell adhesion via temperature‐controlled modulation of ligand density, we investigated the swelling behavior and surface charge of AEMA‐based microgels and their glycan‐functionalized derivatives using dynamic light scattering (DLS) and electrophoretic mobility measurements (Figure 3 and Table 1). The microgels exhibited clear temperature‐responsive behavior, with a pronounced decrease in hydrodynamic diameter upon heating across the VPTT near 32°C, as expected for PNIPAM‐based systems. Previous liquid AFM studies confirm that also adsorbed PNIPAM microgels undergo a strong decrease in volume [8, 41]. The microgels synthesized with 1% or AEMA content were slightly larger than those with 2% AEMA, likely due to slower reaction kinetics and reduced electrostatic repulsion during polymerization. Upon functionalization with glycans, particularly HA and heparin, a substantial increase in particle size was observed at low temperatures, attributed to osmotic swelling from the polyanionic carbohydrate moieties and their associated hydration shells. The most significant swelling‐to‐collapse transition was seen for AEMA2%‐HA microgels, which exhibited a more than twofold size reduction from 891 nm at 20°C to 346 nm at 40°C. This large change suggests a high responsiveness of these particles and points to a strong modulation of HA density on the surface, making them particularly suitable for temperature‐tunable cell adhesion. Importantly, at the onset of the VPTT around 25°C–31°C, a partial size reduction was observed, indicating that meaningful control over ligand density is achievable at biologically relevant temperatures. Furthermore, we can expect that this temperature range is also valid for physiological buffers such as PBS (phosphate buffered saline), as used here. This is because the main constituent NaCl, does not significantly affect the LCST of PNIPAM, as its ions are located in the middle of the Hofmeister series and are considered weak chaotropes [42]. The behavior of AEMA1%‐cGM microgels deviated from the typical trend, with an unexpected size increase at elevated temperatures. This may reflect additional upper critical solution temperature (UCST)‐type swelling behavior associated with the glycan component, and warrants further investigation [43].

Electrophoretic mobility measurements for exemplary conducted for AEMA2%‐and AEMA2%‐HA microgels further confirmed the temperature‐sensitive change in density of ionic groups [44]. Both microgel systems show an increase in mobility upon heating, consistent with the collapse of the microgel network and the concomitant densification of charged units on the particle surface (Figure 3, right). Conversely, lowering the temperature reduces the charge density due to microgel swelling. This reversible change from dense HA units to diffuse HA presentation upon temperature decrease will be central to the mechanism of selective cell release in our assay, as it directly impacts the strength of CD44–HA interactions.

### 2.3 Cell Adhesion/Release Assays

**FIGURE 4:**
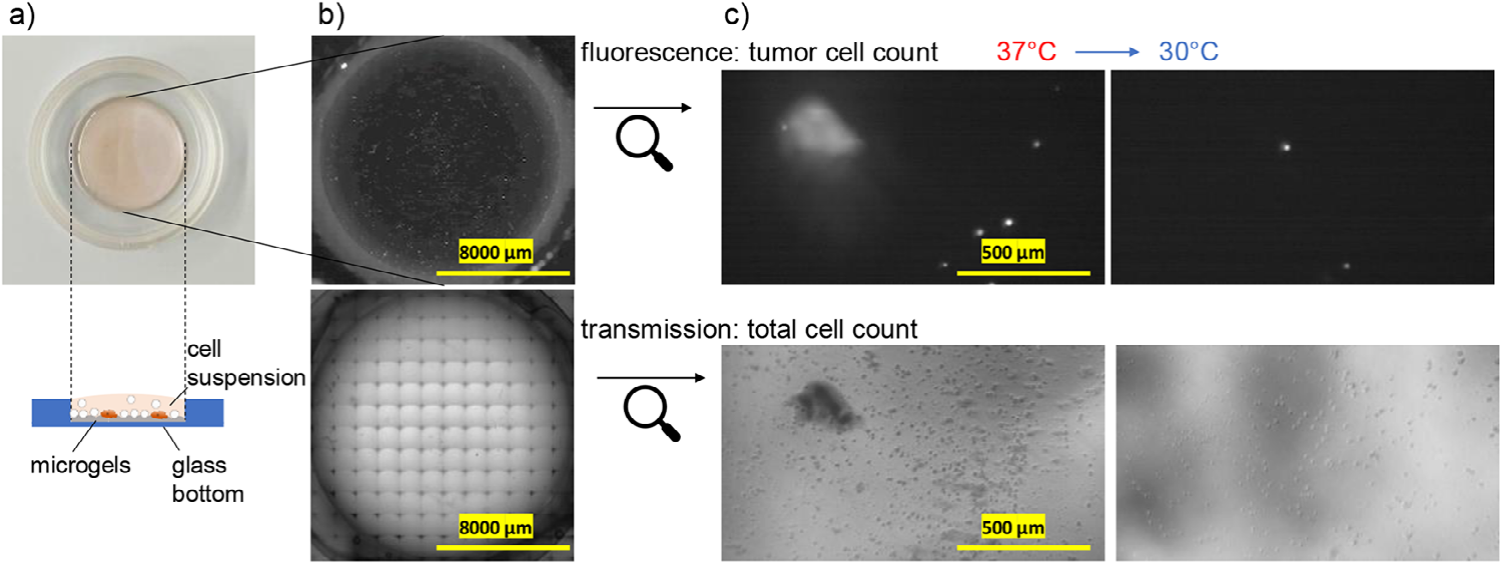
Temperature‐dependent cell adhesion assay using a microgel‐coated dish. (a) Schematic and photograph of the experimental setup: tumor cells are suspended in buffer above a microgel layer on a glass‐bottom dish. (b) Overview images of the cell‐covered microgel surface acquired in fluorescence (top) and transmission mode (bottom) acquired by scanning the whole dish surface. Bright spots signify adhered tumor cells. (c) Representative fluorescence (top row) and transmission images (bottom row) showing tumor cell adhesion (CellTracker Green labeled) and total cell distribution before and after temperature reduction from 37°C to 30°C. A clear decrease in overall cell adhesion is observed upon cooling.

For the cell adhesion assays on the microgel surfaces the tumor cells (MDA‐MB‐231 and CTC‐ITB‐01) were pre‐labeled with CellTracker Green and adjusted to 5000 cells per 500 µL for all experiments. For assays involving blood components, WBCs were isolated from whole blood via erythrocyte lysis and washing steps. Tumor cells were subsequently spiked into the WBC suspension to reach the same final concentration. The cells were then added to the microgel coated round glass bottom culture dishes (Figure 4). Afterward, the cells were incubated for 3 h at 37°C under gentle shaking followed by removal of non‐adhered cells in the supernatant. Next, the entire surface was scanned via fluorescence and transmission microscopy to be able to count and differentiate between tumor cells and WBCs. For cell counting, the area at the periphery of the dish (bright autofluorescence from an adhesive) was excluded from the analysis.

**FIGURE 5:**
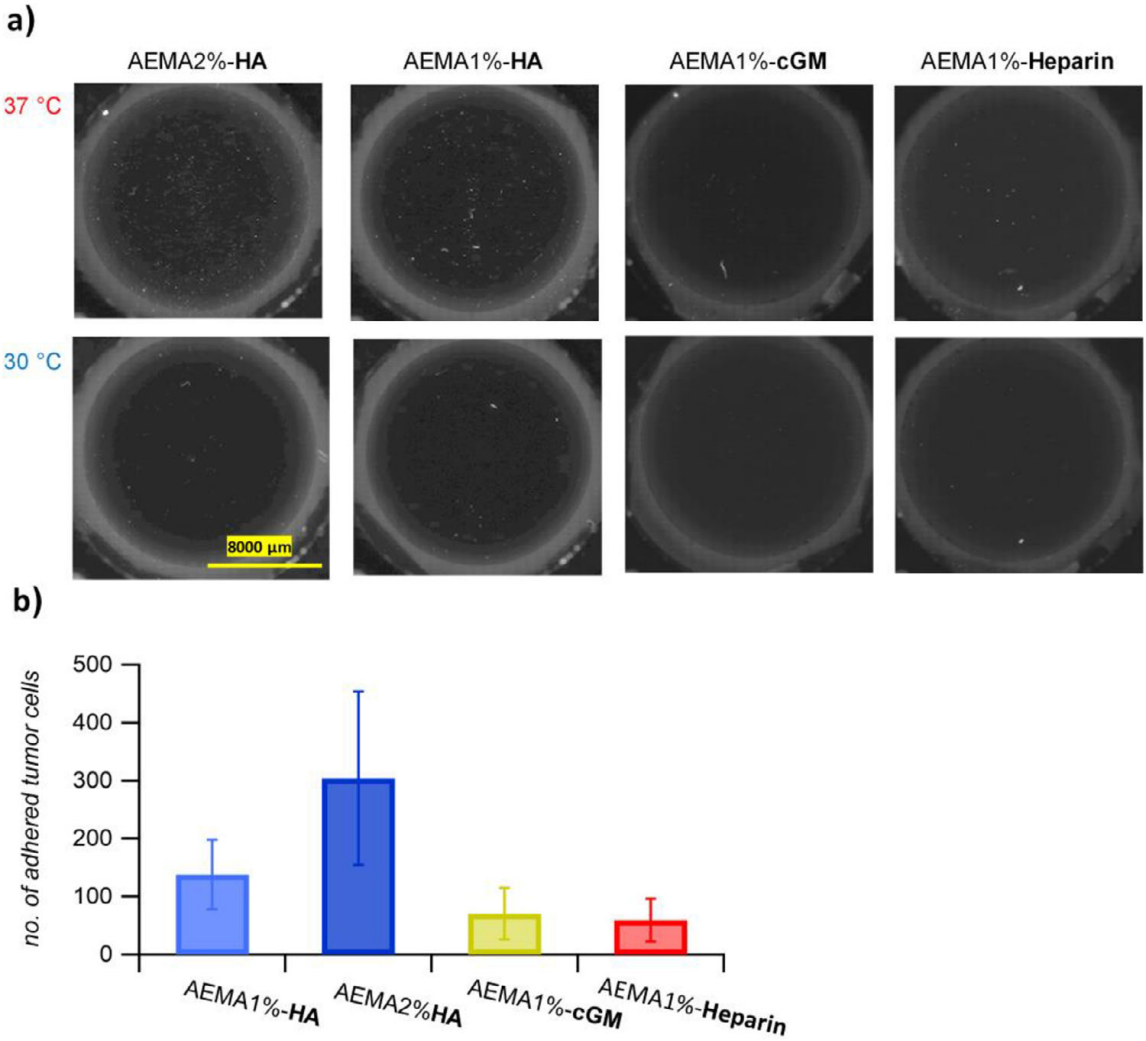
Adhesion of MDA‐MB‐231 tumor cells on microgel coatings at 37°C and 30°C. (a) Fluorescence microscopy images at 37°C (top) and after cooling to 30°C (bottom). Cells appear as small brighter pixels. Strong adhesion is observed on HA‐functionalized coatings, especially with 2 mol% AEMA content. Control surfaces with cGM or heparin show minimal adhesion. (b) Quantification of adhered cells at 37°C. Error bars represent standard deviation from triplicate measurements.

To evaluate the ability of glycan‐functionalized microgel coatings to selectively capture tumor cells, we analyzed the adhesion of fluorescently labeled MDA‐MB‐231 cells on surfaces coated with different microgels. These included Heparin, cGM, and HA‐functionalized microgels with either 1 mol% or 2 mol% AEMA content. At physiological temperature (37°C), strong fluorescence signals indicating cell adhesion were observed significantly stronger on HA‐functionalized surfaces (Figure 5a, top row). In particular, surfaces coated with AEMA2%‐HA microgels showed the highest number of adherent tumor cells, while AEMA1%‐HA coatings supported a moderate number of cells. In contrast, both control surfaces functionalized with cGM or heparin showed only minimal cell adhesion, suggesting that specific CD44–HA interactions are essential to capture MDA‐MB‐231 cells, which are known to express high levels of CD44 [45]. Upon lowering the temperature to 30°C, the number of visible fluorescent spots decreased for all samples (Figure 5a, bottom row), consistent with swelling of the thermoresponsive PNIPAM microgel matrix and a reduction in ligand density. This effect was most pronounced for the AEMA2%‐HA surfaces, supporting the idea that temperature‐triggered changes in polymer conformation effectively modulate ligand presentation and thereby adhesion strength. Quantitative image analysis confirmed these trends (Figure 5b): On average, ∼400 cells adhered to the AEMA2%‐HA surfaces during the first adhesion cycle, whereas fewer than half as many cells adhered to AEMA1%‐HA surfaces. Control coatings showed only minimal capture (<100 cells). Notably, the number of adhered tumor cells remained far below the number of spiked cells (5000), even for the most adhesive surface. This limited yield may be due to the restriction of capture to a single 2D monolayer, lowering the chance multiple cell HA‐surface collisions. The capture could be much more efficient on a larger surface area, e.g., fiber mats or bead suspensions [20, 21, 31, 46]. Additionally, during the adhesion step, samples were placed on a shaker, potentially preventing weakly adhering tumor cells from establishing firm contact with the microgel surface.

In addition to MDA‐MB‐231 cells, we evaluated the adhesion behavior of circulating tumor cells isolated from a metastatic breast cancer patient [47]. When tested on various microgel‐coated surfaces, the cells from the CTC‐ITB‐01 line exhibited weak initial attachment to HA‐functionalized coatings with cell counts comparable to those observed on control surfaces presenting heparin or cGM (Figure S1). Flow cytometry analysis (see Section 2.3) confirmed a low expression of CD44 receptors on these CTCs, providing a plausible explanation for their reduced adhesion to HA‐presenting surfaces.

**FIGURE 6:**
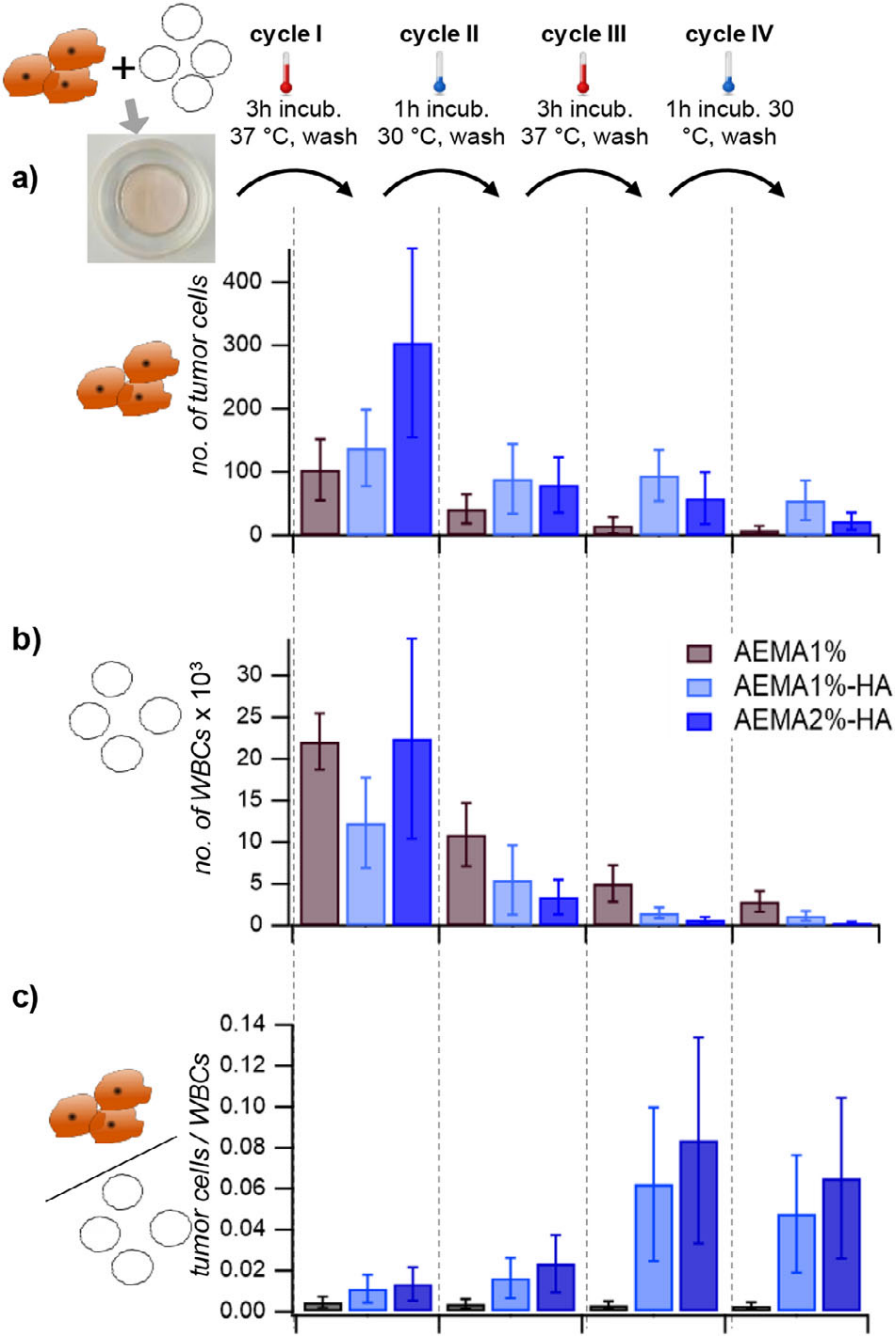
Adhesion and relative enrichment of MDA‐MB 231 breast cancer cells over WBCs on thermoresponsive microgel surfaces across temperature cycles. Tumor cells and WBCs were co‐incubated on microgel‐coated dishes (AEMA1%, AEMA1%‐HA, and AEMA2%‐HA) through sequential incubation, cooling, and washing steps. (a) Quantification of adhered tumor and (b) WBCs cells after each cycle. (c) Ratio of tumor cells to WBCs, showing progressive enrichment of tumor cells HA presenting coatings over repeated temperature cycling. Error bars represent standard deviations of triplicate measurements. Large errors are due variations in starting cell counts between the different surfaces and varying WBC contents in blood.

After establishing the specific adhesion of CD44‐positive tumor cells to HA‐functionalized microgel coatings, we evaluated the ability of these surfaces to selectively retain tumor cells while reducing WBC attachment through temperature cycling under continuous agitation (Figure 6). Cycle I was simply the initial incubation after adding both tumor cells and WBCs on AEMA2%‐HA microgel coatings at 37°C for 3 h. In cycle II the temperature was reduced to 30°C for 1 h, followed by the same heating and cooling cycles (cycles III and IV). Between the different heating and cooling cycles non‐adherent cells were removed by gently washing the surfaces.

As expected, the initial adhesion (cycle I) of tumor cells was somewhat stronger than WBC adhesion, roughly 1/10 of the added tumor cells were found on the surfaces. A much lower fraction of WBC adhered to the surfaces, roughly 1/300 (AEMA‐2%HA) and 1/500 (AEMA‐1%HA) from the 4–11 million cells present in 1 mL of blood [48]. Following a temperature drop to 30°C, swelling of the microgels reduced ligand density and overall surface adhesiveness (cycle II). This led to a marked decrease in adherent cell numbers, particularly among WBCs. Quantitative image analysis revealed that WBC detachment was more pronounced, with cell numbers reduced by a factor of ∼5, compared to a ∼3‐fold reduction for tumor cells. This supports the hypothesis that tumor cells establish stronger, more specific interactions with surface‐bound HA, consistent with their CD44 expression. Interestingly, while the majority of tumor cells remained firmly attached during a subsequent reheating to 37°C combined with prolonged agitation for 3 h (cycle III) resulted in further loss of WBCs. This was somewhat unexpected, given the increased adhesiveness of the surface at elevated temperature. A possible explanation is that the preceding cooling step removed a subpopulation of WBCs capable of stronger HA interactions, leaving behind cells that bound non‐specifically which could not remain attached under prolonged agitation during the reheating phase. Comparison to non‐HA presenting surfaces AEMA, heparin, and cGM showed similar levels of WBC attachment compared to HA surfaces initially (Figure S1), confirming weak WBC‐HA interaction. In contrast, tumor cells likely maintained sufficient residual binding through multivalent CD44–HA interactions. Alternatively, WBCs may lose viability or undergo phenotypic changes that reduce their adhesive potential in subsequent cycles. Therefore, future work shall involve optimizing the microgel's capture efficiency by exploring variables such as incubation time, while this study focuses on a proof‐of‐principle demonstration.

A second cooling step (cycle IV) again reduced overall adhesion, affecting both cell types. However, the relative ratio of tumor cells to WBCs on the surface increased, highlighting the surface's enrichment capability for tumor cells across multiple adhesion/detachment cycles (Figure 6c). Control surfaces functionalized with heparin or cGM failed to show any significant tumor cell enrichment during temperature cycling (Figure S1 confirming that specific HA–CD44 interactions are essential for selective tumor cell retention on the microgel coatings. Overall, these results demonstrate that thermoresponsive HA‐functionalized microgel surfaces enable dynamic control over cell adhesion behavior, selectively enriching MDA‐MB‐231 cells over WBCs in a repeatable and reversible manner.

### 2.4 Flow Cytometry Measurements Confirm that CD44 Overexpressing Cells are Enriched by the Surface

**FIGURE 7:**
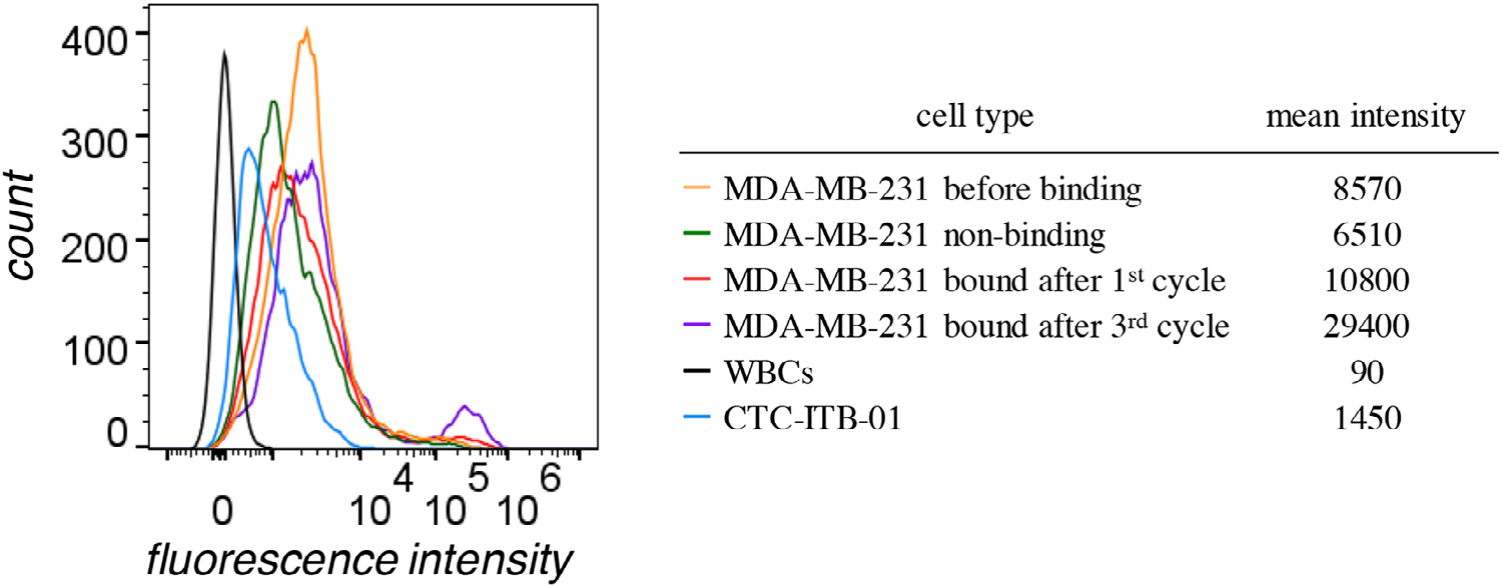
Flow cytometry analysis of CD44 expression in different MDA‐MB‐231 cell sub populations after interaction with HA‐functionalized microgel coatings. CD44 levels in MDA‐MB‐231 cells show enrichment of CD44^high^ cells following surface adhesion. WBCs and CTCs exhibit low CD44 expression, consistent with poor binding to HA surfaces.

Flow cytometry analysis was performed to evaluate the expression levels of CD44 on the various cell populations interacting with the thermoresponsive microgel surfaces. As expected, MDA‐MB‐231 breast cancer cells exhibited a broad CD44 expression profile, and following incubation on HA‐coated microgels, the population of cells binding on the surfaces showed a higher mean CD44 signal as compared to the non‐binding cells (Figure 7a). Importantly, after the prolonged incubation and after the releasing steps the remaining cells showed a progressively stronger CD44 signal. This suggests that the adhesive surface selectively retains CD44^high^ MDA‐MB 231 cells during the cycles. In contrast, WBCs, which expressed significantly lower levels of CD44 (Figure 7b), aligning with their depletion observed in earlier cell adhesion experiments. Interestingly, when the initially detached cell fraction (from cycle II) was reintroduced to the HA‐presenting microgels, no significant further enrichment was observed (Figure S2). This may be attributed to the low number of CD44‐high cells in the population of detached cells. CTC‐ITB‐01 derived from patient samples also exhibited low CD44 expression, consistent with their limited adhesion to HA‐presenting surfaces. These findings underscore the functional selectivity of the surface based on receptor‐ligand interactions to capture CD44^high^ tumor cells. It is important to emphasize the value of enriching CD44^high^ subpopulations as they are thought to represent a cancer stem cell–like phenotype and may play a central role in metastatic dissemination [49]. Consequently, isolating and analyzing this subpopulation could provide highly relevant insights into the tumor cells most critical for disease progression and relapse. Yet, the results also highlight that HA–CD44 binding alone may not suffice for comprehensive CTC capture across heterogeneous phenotypes, warranting combination of further ‘baits’ (capture motifs) such as antibodies. Altogether, these results confirm that thermoresponsive HA‐functionalized microgels can selectively enrich for CD44^high^ tumor cells over WBCs by modulating adhesion through temperature changes. However, optimizing initial binding efficiency and considering alternative HA‐binding receptors or combinatorial capture motifs will be important steps to broaden the applicability of this approach.

## 3 Conclusion and Outlook

In this study, we developed and evaluated coating of thermoresponsive microgels with hyaluronic acid ligands for the selective enrichment of CD44‐expressing tumor cells from complex biological samples. Dynamic light scattering and zeta potential measurements confirmed the temperature‐controlled collapse of the microgel network and corresponding changes in surface charge density, which directly influenced the accessibility and presentation of HA ligands. The biological performance of the coatings was validated in cell adhesion assays using MDA‐MB‐231 breast cancer cells – well known for its high expression of CD44 – and white blood cells (WBCs), demonstrating that only HA‐functionalized microgels enabled strong, selective adhesion of CD44^high^ cells at 37°C. Cooling the system to 30°C caused the microgels to swell and reduced surface ligand density, leading to preferential detachment of weakly adhering WBCs while retaining tumor cells. This temperature‐tuneable adhesion was further confirmed using flow cytometry, which revealed enrichment of CD44^high^ tumor cell subpopulations after interaction with the surface. The system was effective for MDA‐MB‐231 cells, which belong to the triple‐negative breast cancer subtype, for which only few effective capture strategies currently exist. Although the overall recovery rate for MDA‐MB‐231 cells in this system was moderate, it is important to emphasize the value of enriching CD44^high^ subpopulations, as these stem cell–like cells are likely key drivers of metastasis and relapse. In addition, the overall capture efficiency, here limited by the small 2D surface area, suggests that future implementations could benefit from scaling to 3D architectures such as fiber mats or bead suspensions. However, adhesion of tumor cells of a patient‐derived CTC‐ITB‐01 line remained limited, likely due to low CD44 expression [35, 50]. This highlights the importance of adapting ligand density and presentation for capturing more diverse CTC phenotypes.

## 4 Limitation of the Study

In addition to the limitations already discussed, several other aspects should be considered when evaluating the broader applicability of this study. First, while MDA‐MB‐231 cells served as a relevant model, only two cell lines were tested in total, and no functional validation (e.g., proliferation, viability, or molecular profiling post‐enrichment) was performed to assess the quality of the captured cells. Furthermore, flow cytometry and microscopy suggest moderate recovery efficiencies, but the minimal detectable cell number (sensitivity threshold) was not determined. This is especially relevant for clinical use, where CTC concentrations are typically very low. Second, the adhesion‐release cycles were conducted under dynamic shaking conditions, which could lead to detachment of weakly adhering tumor cells, and may therefore reduce recovery. In addition, the platform was only tested using pre‐processed samples (i.e., after erythrocyte lysis and WBC isolation), not in whole blood. This limits insight into matrix‐related interference such as plasma proteins or non‐specific adhesion, and should be investigated. From a translational standpoint, the current system lacks automation and relies on manual microscopy for cell identification and counting. While suitable for proof‐of‐concept, this limits scalability and integration into routine clinical workflows. Similarly, long‐term stability and batch‐to‐batch reproducibility of the HA‐functionalized microgel surfaces were not assessed, which would be essential for clinical standardization. Finally, a direct comparison to established enrichment technologies—such as CellSearch, Parsortix, or antibody‐based magnetic separation—was not included, making it difficult to position the performance of the presented system within the existing diagnostic landscape.

Together, these limitations underscore important opportunities for future development. These include expanding the capture strategy to incorporate additional surface markers, integrating the system with automated imaging and analysis platforms, and validating its performance in whole blood and larger, clinically relevant patient cohorts. Despite current constraints, this study demonstrates that functionalized microgels offer a versatile and synthetically accessible platform for temperature‐responsive modulation of cell adhesion. This approach holds promise for selective cell enrichment, biointerface engineering, and applications in cancer diagnostics.

## 5 Experimental Section

### 5.1 Materials

Ammonium persulfate (APS, 99%, Carbolution), N‐(2 aminoethyl)methacrylamide hydrochloride (AEMA, ≥98%, Sigma–Aldrich), 1 ethyl 3 (3 dimethylaminopropyl)carbodiimde hydrochloride (EDC, >98%, Alfa Aesar), Galactomannan from ceratonia siliqua (cGM, ≥92%, Carbosynth, UK), sodium hyaluronate 8 000 15 000 g∙mol^−1^ (HA, ≥91%, Carbosynth), N isopropylacrylamide (NIPAM, 99%, Acris Organics), N hydroxysuccinimide (NHS, 98%, Fluorochem), N,N methylenbisacrylamide (MBA, ≥98%, Alfa Aesar), n octadecyltrichlorosilane (95%, ABCR), DAKO protein block serum free (Dako Corporation), Dulbecco´s phosphate‐buffered saline (Gibco), fetal calf serum (Gibco), penicillin streptomycin 10 000 U/mL (Gibco), RPMI‐1640 medium L‐glutamine (Gibco), trypsin (Gibco), CellTracker Green CMFDA Dye (Invitrogen)

### 5.2 Microgel Preparation

*N*‐isopropylacrylamide (12 mmol), *N*‐(2‐Aminoethyl)methacrylamide hydrochloride (0.12 mmol for 1mol% and 0.24 mmol for 2mol%), *N,N’*‐methylenebisacrylamide (0.7 mmol) were dissolved in 148 mL ultrapure water. The solution was heated to 70°C for 30 min with N_2_ purge and stirring. To initiate the reaction, ammonium persulfate (0.05 mmol) dissolved in 2 mL water was added and stirred for 150 min under N_2_ purge. The reaction was stopped by cooling down to room temperature. For purification, the microgels were centrifuged several times and later freeze‐dried. The microgels were functionalized with the polysaccharides via the amine group of the comonomer AEMA via carbodiimide chemistry in MES (2‐(N‐morpholino)ethanesulfonic acid) buffer (10 mm MES, pH 5). For AEMA1%‐HA, AEMA1%‐cGM, AEMA1%‐heparin microgels, 20 mg of the microgels were dissolved in 4 mL MES buffer. 175 mg of the polysaccharides were dissolved in 3 mL MES buffer and added to the microgel suspension, followed by adding NHS (152 µmol) in 1 mL MES buffer, EDC (152 µmol) in 0.333 mL, and reacting overnight at room temperature on a shaker. For AEMA2%‐HA microgels the reagent concentrations were doubled. The hydrodynamic radii and the zeta potentials were measured in water at a concentration of 0.05 mg·mL^−1^ and 1 mm HEPES (4‐(2‐hydroxyethyl)‐1‐piperazineethanesulfonic acid) buffer at pH 7.4 on a Zetasizer Nano Series Nano ZS (Malvern GmbH, Germany).

### 5.3 Quantification of Functional Groups

To determinate the amount of amine groups of the microgels before coupling of HA, the Kaiser Test were performed. 1 mg of the microgel was dissolved in 1667 µL ethanol. 111 µL reagent A (0.01 m potassium cyanide solution in pyridine), 111 µL reagent B (50 g/L ninhydrin in ethanol), and 111 µL reagent C (2 g/mL phenol in ethanol) were added to the solution and mixed well. The sample was heated 5 min at 100°C in an oil bath and cooled afterward for 20 min at room temperature. The solution was centrifuged for 10 min at room temperature and 13 000 rpm. The supernatant was used for the following measurement. The samples were analyzed via UV‐Vis‐spectroscopy at the range of 400–650 nm. The concentration of the amines in the microgel could be determined using a previously created calibration curve via the absorbance value at 570 nm.

The Phenol‐sulfuric acid method was performed to determinate the amount of carbohydrates in the microgels. Therefor 1 mg of the sample was dissolved in 250 µL ultrapure water. 250 µL of 5% phenolic solution and 1250 µL concentrated sulfuric acid were added and mixed well. Due to the addition of concentrated sulfuric acid the solution heated up. The sample was incubated at room temperature for 20 min. Afterward the solution was centrifuged for 10 min at room temperature and 13 000 rpm. The supernatant was used for the following measurements. The samples were analyzed via UV–Vis‐spectroscopy at the range of 400–550 nm. To determine the total amount of carbohydrates in the sample, the value of absorbance at 490 nm was used for the calculation of a previously created calibration curve.

The TBO (toluidine blue O) test was carried out to determine the amount of carboxyl groups of the carboxylated GM. For this purpose, 1 mL of a 40‐fold diluted TBO solution (12.5 mm, pH 10.3) was added to 1 mg of the sample in an Eppendorf tube and shaken overnight in the dark at room temperature. The samples were centrifuged for 10 min at 13 000 rpm, and then 0.3 mL of the supernatant was taken, and the volume was adjusted to 2 mL with MiliQ water (pH 10.3). To analyze the amount of carboxyl groups the samples the absorbance at 633 nm was analyzed via UV‐Vis‐spectroscopy to obtain the amount of carboxyl groups per g sample (absorbance was calibrated with known carboxyl groups content).

### 5.4 Surface Preparation

Glass Bottom culture dishes (35 mm, ibidi, Germany) were used to coat the microgel surfaces. First were treated with a UV ozone cleaner UVC 1014 (NanoBioAnalytics, Berlin, Germany) for 30 min. Subsequently, the dishes were coated via vapor deposition of octadecyl trichlorosilane at 0.25 mbar in the desiccator for 24 h. Afterward, the dishes were rinsed with isopropanol, dried, and coated with 1.5 mL of microgel dispersion (31.25 µg/mL). The dishes were dried at 40°C in a drying oven until the liquid was removed. Later, 1 mL ultrapure water was added to the plates and shaked over night to remove possible multilayers. To remove excess microgel, the plates were washed several times with Ultrapure water and dried again. The microgel films were imaged in water by optical microscopy in reflection mode or in dry state using an atomic force microscope (AFM, Nanowizard IV, Bruker, Berlin, Germany) in intermittent contact mode using cantilevers with a nominal spring constant of 300 N m^−1^ (HQ:XSC11, MikroMash, Bulgaria).

### 5.5 Cell Cultivation and Flow Cytometry Analysis

MDA‐MB‐231 (obtained from the American Type Culture Collection, USA) cell line was grown in RPMI 1640 medium with 10% FBS (fetal bovine serum) and 1% P/S (penicillin‐streptomycin). CTC‐ITB‐01 cell line was grown in RPMI 1640 medium with 10% FBS, 1% P/S, 1% L‐glutamine, 1% Insulin‐Transferrin‐Selenium‐A Supplement, 10 ng/mL FGF2, 50 ng/mL EGF, 0.1 lg/mL hydrocortisone, and 0.2 lg/mL cholera toxin. Cells were harvested with enzyme‐free cell dissociation solution. To obtain WBCs, 5 mL peripheral blood from healthy donors (GynBiobank, approved by Heinrich Heine University Duesseldorf, Ref.‐Nr. 2020‐866‐bio) were collected in EDTA (ethylenediaminetetraacetic acid) tubes and added to 15 mL BioColl separation solution in SepMate tubes. The sample was centrifuged at 1200g for 10 min at RT. The upper part was transferred to a 15 mL falcon. The falcon was filled up to 15 mL with PBS and centrifuged. The pellet (WBCs) was washed with PBS and used for experiments. For flow cytometry labelling, the cells were incubated with CD44 antibody (HPA005785, 1:300) for 1 h at 4°C. After washing with PBS, the samples were incubated with donkey anti‐rabbit IgG (H&L) Alexa Fluor 594 (1:1000) for 1 h at RT, then washed and analyzed with CytoFLEX S flow cytometry (Beckman Coulter). The FACSDiva software (BD) was used for data evaluation.

### 5.6 Cell Adhesion Assay

Cancer cells were labeled by incubating them with CellTracker Green. Therefore, 1 µL CellTracker Green solution was added to 1 mL cell solution and incubated for 30 min at 37°C in the incubator. The cells were then centrifuged 3 times, and the supernatant was removed and replaced with PBS buffer. The cells were then counted using a Neubauer chamber, and the concentration was adjusted to always add 5000 cells in 500 µL buffer to each microgel dish before the measurement. For the measurements, the buffer was replaced by PBS, and MDA‐MB‐231 cells or CTC‐ITB‐01 cells were spiked into the WBC solution to attain a concentration of 5000 cells in 500 µL. Cell suspensions were added to microgel‐coated, glass‐bottom dishes and incubated for 3 h at 37°C under gentle agitation (cycle I, cycle II) or for 1 h at 30°C (cycle II, IV) as given in the main text. Following incubation, non‐adherent cells were removed, and the entire surface was imaged by fluorescence and transmission microscopy on an Olympus IX73 equipped with a scanning stage and a heating stage (Petri dish heater, JPK, Berlin) to quantify adherent tumor cells and distinguish them from WBCs.

## Conflicts of Interest

The authors declare no conflicts of interest.

## Supporting information




**Supporting File**: marc70058‐sup‐0001‐SuppMat.docx.

## Data Availability

The data that support the findings of this study are available from the corresponding author upon reasonable request.
