# Supplementary material for: Enrichment of Hyaluronic Acid Binding Tumor Cells by Modulation of Selective Adhesion on Microgel Surfaces
Source: Macromol Rapid Commun. 2025 Sep 24;47(14):e00549. doi: 10.1002/marc.202500549 (PMC13384807; doi:10.1002/marc.202500549)
Supplement: Supplementary file 1 — Supporting File: marc70058‐sup‐0001‐SuppMat.docx. [file MARC-47-e00549-s001.docx]

# Supporting information

Enrichment of hyaluronic acid binding tumor cells by modulation of selective adhesion on microgel surfaces

Melanie Schmidt, Dilay Karayel, André Franken, Janita Müller, Laura Hartmann, Klaus Pantel, Tanja Fehm, Hans Neubauer* and Stephan Schmidt*

Hans Neubauer, Dilay Karayel, André Franken, Tanja Fehm

Department of Obstetrics and Gynecology, University Hospital and Medical Faculty of the Heinrich-Heine University Duesseldorf, Moorenstr. 5, 40225, Düsseldorf, Germany.

Center for Integrated Oncology (CIO Aachen, Bonn, Cologne), Duesseldorf, Germany.

M. Schmidt, Janita Müller

Heinrich- Heine- University Düsseldorf, Faculty of Mathematics and Natural Sciences, Institute of Organic Chemistry and Macromolecular Chemistry, 40204 Düsseldorf, Germany

Klaus Pantel

Department for Tumour Biology, University Medical Centre Hamburg-Eppendorf,

Martinistraße 52, 20246, Hamburg, Germany.

S. Schmidt, Laura Hartmann

Institute for Macromolecular Chemistry, Faculty of Chemistry and Pharmacy, Albert-Ludwigs-Universität Freiburg, 79104 Freiburg, Germany

E-mail:

stephan.schmidt@makro.uni-freiburg.de

Hans.Neubauer@med.uni-duesseldorf.de

Supporting information S1: Additional cell adhesion data for heparin, galactomann coated microgel surfaces


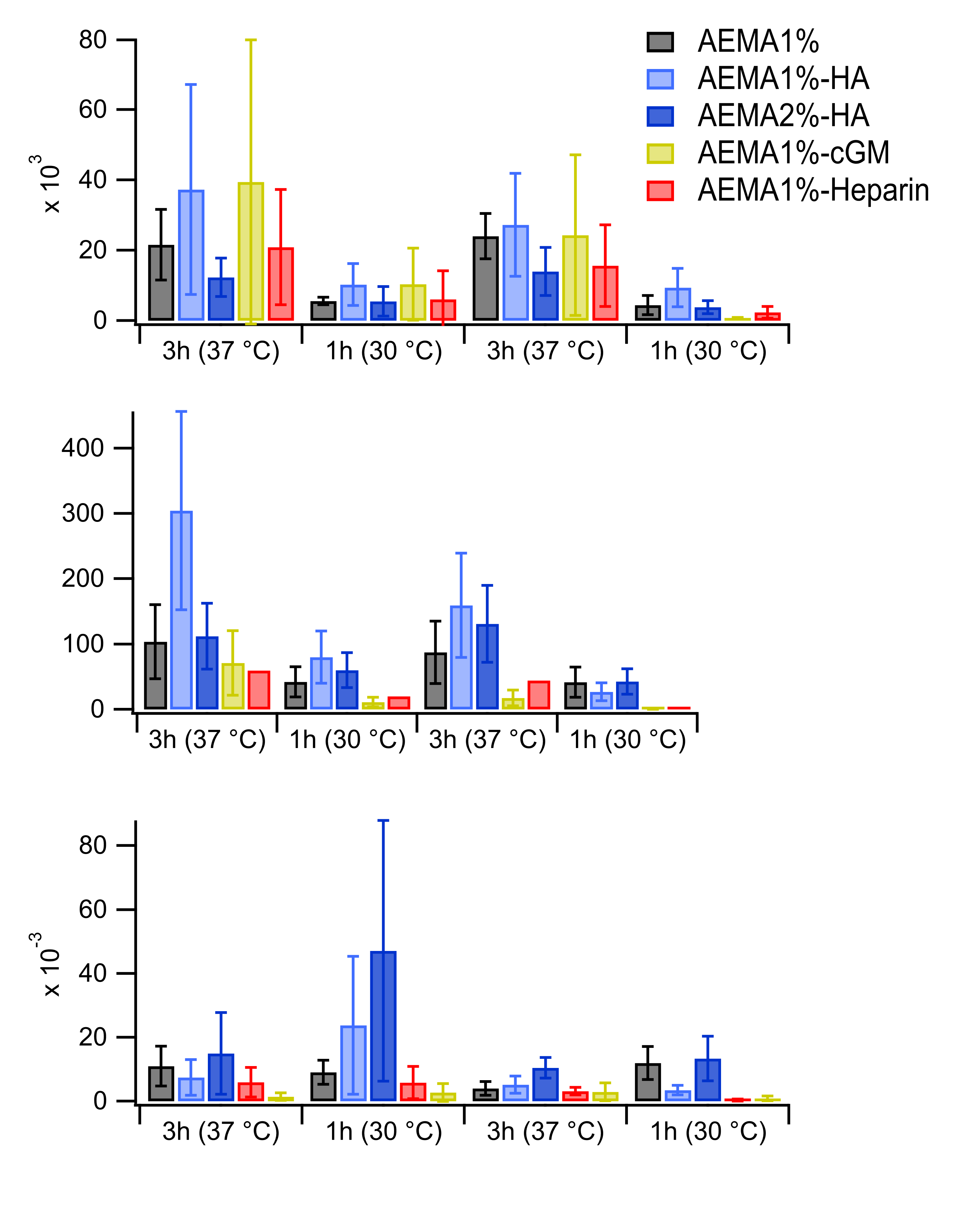


Figure S 1 Additional cell adhesion data for heparin, galactomann coated microgel surfaces

Supporting information S2: Cell adhesion of reintroduced cells


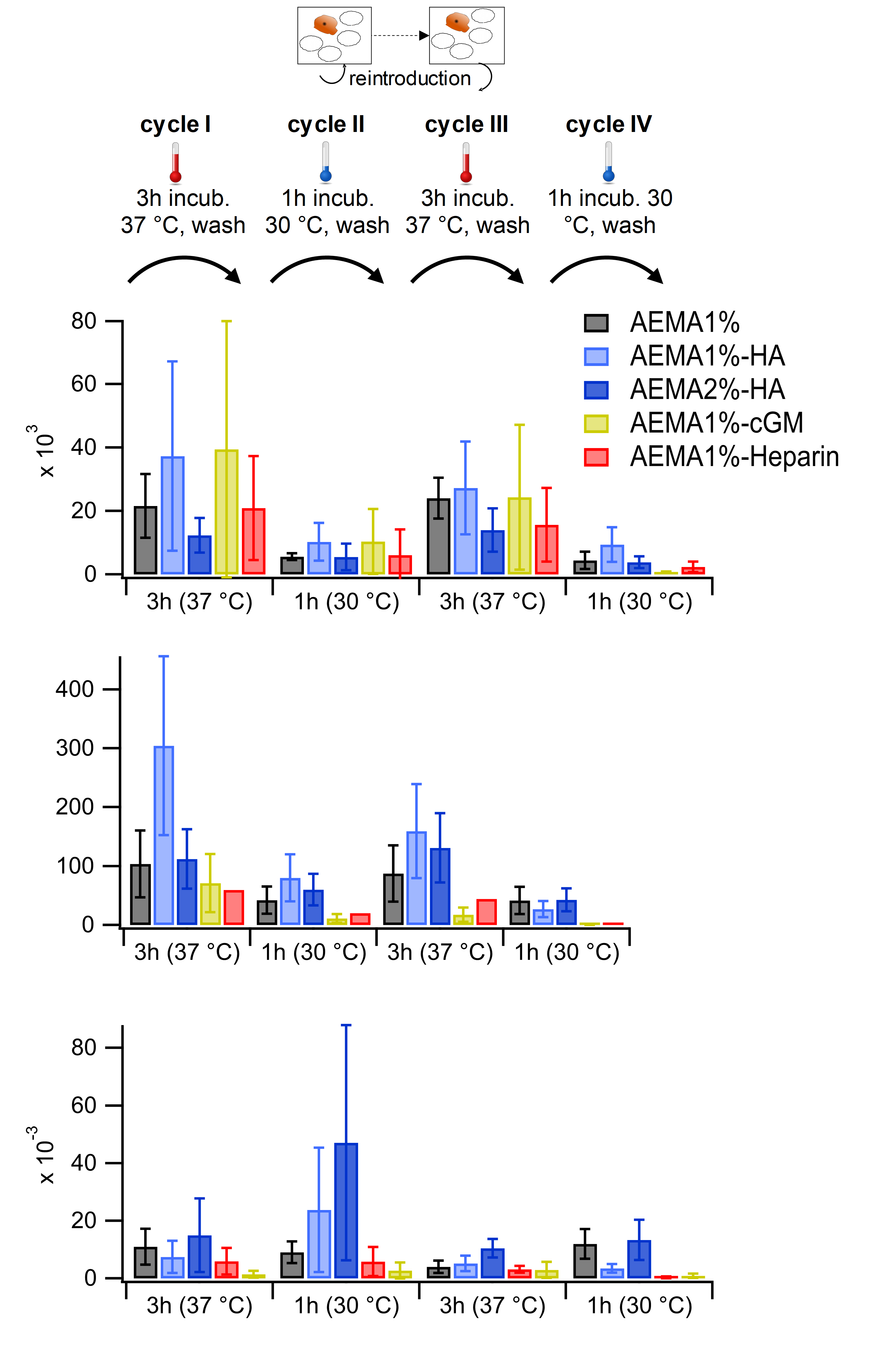


Figure S 2 Cell adhesion assay with reintroduced cells
